# Association of Marijuana Legalization With Marijuana Use Among US High School Students, 1993-2019

**DOI:** 10.1001/jamanetworkopen.2021.24638

**Published:** 2021-09-07

**Authors:** D. Mark Anderson, Daniel I. Rees, Joseph J. Sabia, Samuel Safford

**Affiliations:** 1 Department of Agricultural Economics and Economics, Montana State University, Bozeman; 2 Department of Economics, Universidad Carlos III de Madrid, Madrid, Spain; 3 Center for Health Economics and Policy Studies, San Diego State University, San Diego, California

## Abstract

This cross-sectional study assesses the association between US state legalization of medical and recreational marijuana use and use of marijuana by adolescents.

## Introduction

Thirty-six states have legalized medical marijuana and 18 states have passed recreational marijuana laws (RMLs). Organizations such as the American Academy of Pediatrics are concerned that legalization will encourage youth marijuana use.^1^ Marijuana use during adolescence may adversely affect areas of the prefrontal cortex, which control important cognitive processes.^1^

Using data from the Youth Risk Behavior Survey (YRBS) for the period 1993-2017, Anderson et al^2^ found that RML adoption was associated with an 8% decrease in the odds of marijuana use among high school students. These authors, however, had prelegalization and postlegalization data from only 7 states and pre– and post–recreational sales data from only 3 states, calling into question the generalizability of their results.^3^

Using data from the YRBS for the period 1993-2019, this study provides updated estimates of the association between legalization and adolescent marijuana use. During this extended period, pre- and post-RML data from the YRBS are available from 10 states; 7 states contributed more than one wave of post-RML data, and these same 7 states contributed data to the YRBS before and after the first dispensary sales began. Anderson et al^3^ had multiple waves of post-RML data from 3 states.

## Methods

Following Anderson et al,^2^ national and state YRBS data were pooled in this repeated cross-sectional study. Ethical review was not required because analyses of secondary, deidentified data are considered exempt from requiring institutional review board approval by the San Diego State University Institutional Review Board. The study followed the Strengthening the Reporting of Observational Studies in Epidemiology (STROBE) reporting guidelines.

Multivariable logistic regression was used to estimate the association between legalization and marijuana use. Effective legalization dates come from Anderson and Rees.^4^ To control for time-invariant factors at the state level and common trends, all models were adjusted for 50 state and 13 survey wave indicators. Alternative models were further adjusted for individual- and state-level characteristics; event-study estimates were produced by replacing the RML indicator with a series of leads and lags. Two-sided hypothesis tests were used, and estimates were considered significant if *P* < .05. Analyses were conducted with STATA 16.1 (StataCorp).

## Results

The mean (SD) age of YRBS respondents was 15.9 (1.23) years, 51.4% of respondents reported as female, and 57.6% reported as non-Hispanic white. The first two columns of the Table show estimated odds ratios (ORs) of current and frequent marijuana use, adjusted for state and survey-wave indicators. In the third and fourth columns, ORs were further adjusted for individual- and state-level covariates.

**Table.** Logistic Estimates of the Association of Marijuana Legalization With Adolescent Marijuana Use^a^

Variable	OR (95% CI)			
	Model 1^b^		Model 2^c^	
	Current use	Frequent use	Current use	Frequent use
**National and state YRBS (n = 1 610 605)**				
RML	1.02 (0.91-1.15)	1.01 (0.90-1.13)	1.00 (0.92-1.10)	0.98 (0.90-1.07)
MML	0.95 (0.91-1.00)	0.95 (0.89-1.01)	0.94 (0.89-0.98)^d^^,^^e^	0.93 (0.87-0.99)^d^
**National YRBS (n = 191 923)**				
RML	0.90 (0.76-1.05)	0.87 (0.68-1.11)	0.86 (0.73-1.01)	0.83 (0.66-1.04)
MML	0.83 (0.71-0.98)^d^	0.76 (0.59-0.98)^d^	0.84 (0.73-0.98)^d^	0.78 (0.60-0.98)^d^
**State YRBS (n = 1 418 682)**				
RML	1.04 (0.92-1.17)	1.03 (0.92-1.15)	1.02 (0.93-1.13)	1.01 (0.93-1.10)
MML	0.97 (0.93-1.01)	0.97 (0.92-1.03)	0.96 (0.91-1.00)	0.96 (0.90-1.02)
**National and state YRBS, replacing RML with RML sales allowed (n = 1 610 605)**				
RML sales allowed	0.89 (0.79-1.00)^d^	0.95 (0.79-1.15)	0.88 (0.79-0.98)^d^	0.93 (0.79-1.10)
MML	0.95 (0.90-0.99)^d^	0.95 (0.89-1.01)	0.93 (0.89-0.98)^d^	0.93 (0.87-0.99)^d^

Abbreviations: MML, medical marijuana laws; OR, odds ratio; RML, recreational marijuana laws; YRBS, Youth Risk Behavior Survey.

^a^ Each column within each panel reports unweighted estimates from a separate logistic regression based on biennial data from the YRBS (1993-2019). Specifically, ORs of current marijuana use (ie, any use in the past 30 days) and frequent marijuana use (ie, use at least 10 times in the past 30 days) are reported. Standard errors, which were used to construct the 95% CIs, were corrected for clustering at the state level.

^b^ Estimated ORs were adjusted for 50 state and 13 survey wave (ie, year) indicators.

^c^ Estimated ORs were adjusted for individual-level characteristics (age, sex, grade, and race), whether marijuana use and possession were decriminalized in the respondent’s state, the presence of a state-level 0.08 blood alcohol concentration law, the state beer tax, state income per capita, state unemployment rate, and 50 state and 13 survey wave indicators.

^d^ Statistically significant at *P* < .05.

^e^ Statistically significant at *P* < .01.

Based on the pooled YRBS data, and in the fully adjusted models, RML adoption was not associated with current marijuana use (OR, 1.00; 95% CI, 0.92-1.10) or frequent marijuana use (OR, 0.98; 95% CI, 0.90-1.07). In the fully adjusted models, medical marijuana law (MML) adoption was associated with a 6% decrease (OR, 0.94; 95% CI, 0.89-0.98) in the odds of current marijuana use and a 7% decrease (OR, 0.93; 95% CI, 0.87-0.99) in the odds of frequent marijuana use. Estimates from separate analyses of the national and state YRBS and estimates of the association between the opening of the first recreational dispensary and marijuana use were qualitatively similar to those above (Table).^5^

Finally, the Figure shows RML event-study estimates (Panel A). Prior to legalization, there was no association with marijuana use, suggesting the parallel-trends assumption held. After 2 or more years, RML adoption was associated with a decrease in marijuana use (OR, 0.85; 95% CI, 0.76, 0.95). Panel B of the Figure shows an event study for MML adoption.

**Figure.**
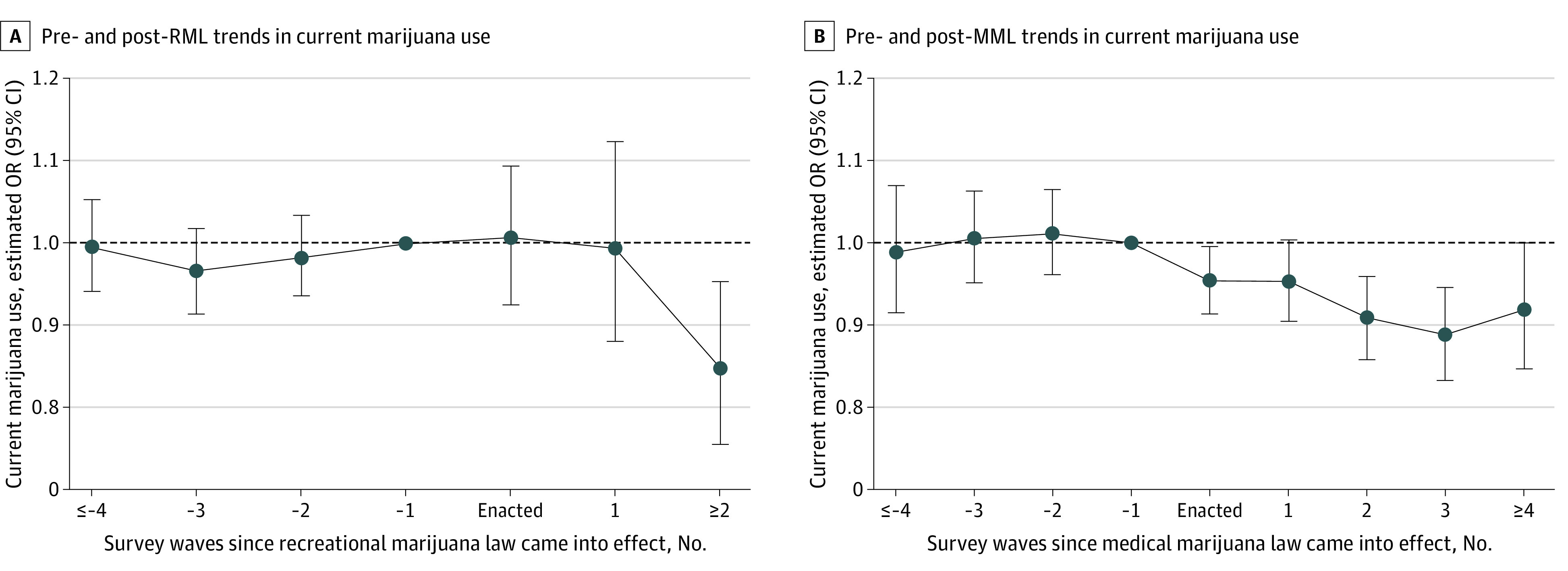
Event Study Analysis Each panel reports unweighted estimates from a separate logistic regression based on biennial data from the national and state Youth Risk Behavior Surveys (1993-2019) including 1 610 605 participants. Specifically, estimated odds ratios (ORs) of current marijuana use (and their 95% CIs) are reported. ORs were adjusted for individual-level characteristics (age, sex, grade, and race), whether marijuana use and possession were decriminalized in the respondent’s state, the presence of a state-level 0.08 blood alcohol concentration law, the state beer tax, state income per capita, state unemployment rate, 50 state and 13 survey wave (ie, year) indicators. The omitted category was 1 survey wave prior to legalization going into effect.

## Discussion

Consistent with estimates from prior studies, there was little evidence that RMLs or MMLs encourage youth marijuana use.^2,6^ Contrary to results of the study by Anderson et al^2^ the overall association between RML adoption and marijuana use among adolescents was statistically indistinguishable from zero. One limitation of this study is that RMLs are a relatively new phenomenon. As more postlegalization data become available, researchers will be able to draw firmer conclusions about the relationship between RMLs and adolescent marijuana use.
